# Detecting healthcare-associated transmission and antifungal resistance in *Candida auris* via whole genome sequencing

**DOI:** 10.1128/jcm.01348-25

**Published:** 2026-03-25

**Authors:** Shannon G. Murphy, Tracy Ross, Anna Fitzgerald, Nick P. G. Gauthier, Eric Keller, Erin Barker, Warda Memon, Samuel D. Chorlton, Terence Moore, Melanie S. Curless, Valeria Fabre, Lisa L. Maragakis, Patricia J. Simner, Sean X. Zhang

**Affiliations:** 1 Department of Pathology, Johns Hopkins University School of Medicine 1500 , Baltimore, Maryland, USA; 2 Microbiology Laboratory, The Johns Hopkins Hospital 588543, Baltimore, Maryland, USA; 3 BugSeq Bioinformatics Inc., Vancouver, British Columbia, Canada; 4 Maryland Department of Health Laboratories Administration 644518 , Baltimore, Maryland, USA; 5 Department of Medicine, Division of Infectious Diseases, Johns Hopkins University School of Medicine 1500 , Baltimore, Maryland, USA; 6 Department of Hospital Epidemiology and Infection Control, The Johns Hopkins Hospital 588543, Baltimore, Maryland, USA; 7 Division of Clinical Microbiology, Department of Laboratory Medicine and Pathology, Mayo Clinic 6915, Rochester, Minnesota, USA; University of Utah, Salt Lake City, Utah, USA

**Keywords:** hospital-associated infection, strain typing, epidemiology, sequencing, *Candida auris*

## Abstract

*Candida auris* is an emerging pathogen known for causing invasive, multidrug-resistant infections and healthcare-associated outbreaks. Measuring the relatedness of *C. auris* strains is critical for detecting and preventing healthcare-associated transmission. Whole genome sequencing (WGS) and analysis of single-nucleotide polymorphisms (SNPs) offer high-resolution assessments of genetic relatedness and antifungal susceptibility; however, interpretation requires an understanding of inter- and intra-patient *C. auris* diversity and genotypic markers of resistance, respectively. This study aimed to define SNP thresholds for assessing *C. auris* relatedness in healthcare-associated outbreak investigations, predict antifungal resistance via genotypic markers, and evaluate patient risk factors to inform future surveillance strategies. WGS was performed on 68 *C. auris* isolates (clades I and III) obtained from 31 hospitalized patients across a healthcare system between 2021 and 2024. Using MycoSNP analysis, we observed a maximum intra-patient variation of 14 SNPs. Five probable transmission clusters were identified based on epidemiologic links, with patient isolates differing by a median of 5 SNPs (range: 0−12). Analysis with a commercial pipeline (refMLST, BugSeq) showed similar clustering patterns. All new detections occurring more than 1 month after admission were linked to a cluster, representing a highly specific indicator of healthcare-associated infection. WGS detected known genotypic markers for fluconazole (ERG11 Y132F, F126L, and MRR1 N647T) and micafungin (FKS1 F635C) resistance, with the latter emerging during antimicrobial therapy. In addition, putative FUR1 mutations (G207R and Q16::STOP) associated with flucytosine resistance were identified. These findings emphasize the utility of WGS for identifying healthcare-associated clusters of *C. auris* and predicting antifungal resistance.

**IMPORTANCE:**

*Candida auris* is a difficult-to-treat yeast that causes invasive infections in vulnerable patient populations. Healthcare exposure is a key risk factor for becoming colonized or infected with *C. auris*, and infection prevention groups focus on curbing the spread of this organism within the healthcare environment. Whole genome sequencing approaches are key for supporting these efforts, as they can help define clusters of *C. auris* transmission and can also provide insight into antifungal resistance. Our work provides practical guidance for interpreting genomic data in this setting, helping infection prevention teams respond more effectively to outbreaks and expanding the use of genotypic predictions for antifungal resistance.

## INTRODUCTION

*Candida auris* is an opportunistic pathogen and an emerging threat in healthcare settings worldwide. This yeast causes invasive infections with high mortality rates in critically ill and immunocompromised patients (1), and treatment is often complicated by resistance to multiple antifungal classes (2). The transmission dynamics of *C. auris* present additional challenges for healthcare facilities, as it readily colonizes the skin and spreads person-to-person (3). *C. auris* can also form biofilms and tolerate common disinfectants (4), facilitating transmission via surfaces or medical equipment. For these reasons, healthcare exposure increases the risk of *C. auris* colonization and infection.

Outbreak investigations rely on both epidemiological (e.g., shared space, equipment, and personnel) and laboratory data; however, conventional methods for detecting and identifying *C. auris* do not typically assign clades or assess genetic similarity (5). Various strain typing methods have been used to assess relatedness during investigations, including DNA fingerprinting (e.g., pulsed-field gel electrophoresis), targeted DNA sequencing (e.g., multi-locus sequencing typing), and whole genome sequencing (WGS) (6–12).

WGS provides high-resolution data for outbreak investigations by quantifying single-nucleotide polymorphisms (SNPs) and other allelic differences (e.g., insertion and deletions) (13). Interpretive thresholds that categorize organisms as “closely” or “not closely” related may be impacted by genome size, mutation rate, host factors (e.g., duration of colonization and selective pressures), and bioinformatic tools used (14). Establishing interpretive thresholds involves assessing maximum SNP distances within individuals (i.e., intra-patient variation) and between epidemiologically linked and unlinked patients (inter-patient variation) (15). Beyond relatedness analyses, WGS offers insight into antifungal resistance by detecting known genotypic markers or facilitating novel discovery through paired phenotypic data.

In this study, we aimed to (i) define SNP thresholds for assessing *C. auris* relatedness for outbreak investigations using open-source and commercial bioinformatic pipelines, (ii) identify genotypic markers of antifungal resistance, and (iii) evaluate the risk factors within our patient population to inform future *C. auris* surveillance strategies.

## MATERIALS AND METHODS

### *C. auris* isolates

We retrospectively and prospectively collected 68 *C. auris* strains from 31 unique patients hospitalized at the Johns Hopkins healthcare system (including the Johns Hopkins Hospital and four regional affiliate hospitals) between 2021 and 2024 (Fig. S1A). Isolates were derived from various clinical specimens (e.g., blood, urine, respiratory, tissue, fluid, etc.), and a limited number was obtained from the axilla/groin as part of point prevalence surveillance (Fig. S1B). Organism identification was performed using MALDI-TOF MS on the Bruker MALDI Biotyper CA System (Bruker, Billerica, MA) with a database that includes spectra for *C. auris* (MBT-CA 3.2.14 library, claim 4 or later).

### Medical record review

Patient medical records were reviewed for demographic information, hospital course, and risk factors for *C. auris* colonization or infection. Infection preventionists reviewed cases for epidemiological links (e.g., temporal/spatial overlap, shared equipment, and personnel). Transmission clusters were assigned based on a combination of epidemiologic and/or strain typing data.

### Whole genome sequencing

DNA was extracted using Quick-DNA Fungal/Bacterial MiniPrep Kit (Zymo, Irvine, CA, D6005) and prepared using Illumina DNA Preparation kits (Illumina, San Diego, CA, 20060059) and indices (Illumina, San Diego, CA, 20027214), according to the manufacturer’s instructions. Libraries were pooled and sequenced using a 300-cycle cartridge and flow cell for either NextSeq (Illumina, San Diego, CA, 20046813) or MiSeq (Illumina, San Diego, CA, MS-102-2002). The median genome depth was 218× (range: 27×–962× for individual isolates) (Fig. S1C).

### Bioinformatic analysis

Paired-end Fastq files were uploaded to BugSeq (Vancouver, British Columbia) for analysis using two different bioinformatic pipelines. The primary analysis method was MycoSNP, developed by the Centers for Disease Control and Prevention, which uses masked, clade-specific *C. auris* reference strains (Clade I, GCA_016772135.1; Clade III GCF_002775015.1) and identifies SNPs using GATK variant (16). The secondary analysis method was BugSeq’s reference-based Multilocus Sequence Typing (refMLST) method, which aligns *de novo* assemblies of queried samples against the NCBI RefSeq reference genome for *C. auris* (17) and quantifies allelic differences (i.e., SNPs and insertions/deletions). Output distance matrices from each pipeline were compared and assessed in the context of epidemiologic data. Trees were constructed using Microreact (Fig. S1D) (18). Clade assignments and detection of genotypic resistance markers were included as part of the BugSeq-supported analyses, which offered a curated database of resistance markers.

### Phenotypic antifungal susceptibility testing

Minimum inhibitory concentrations (MICs) were measured using Sensititre YeastOne panels (ThermoFisher Scientific, Waltham, MA, YO9). Amphotericin B MICs were measured by gradient diffusion (bioMérieux, Marcy l’Étoile, France, 424317) on RPMI agar. Resistance interpretations were based on proposed CDC cutoffs: fluconazole (≥32 µg/mL), micafungin (≥4 µg/mL), and amphotericin B (≥2 µg/mL) (19). Elevated MICs for flucytosine were defined as ≥32 µg/mL.

### Statistical analysis

Analyses were performed using GraphPad Prism software version 10.0.0 (GraphPad, Boston, MA). Data normality was assessed using the Shapiro-Wilk test. Mann-Whitney and Kruskal-Wallis tests were used to compare two independent groups and pairwise differences, respectively.

## RESULTS

### Demographics and clinical characteristics of patients colonized or infected with *C. auris*

Characteristics of 31 patients with *C. auris* are presented in Table 1. The median age at first *C. auris* detection was 59 years (range: 1–89), and 65% of patients were men. Thirty-five percent of patients had *C. auris* recovered from multiple sites, most commonly blood (42%), respiratory (29%), and urine (29%). Most (68%) patients had exposure to healthcare settings—either residence at a skilled nursing or long-term acute care facility (50%) or transfer from another hospital (18%). Two patients (6.5%) had international healthcare exposure. Most patients required admission to an intensive care unit (58%) or use of invasive medical devices, including central venous catheters (74%), indwelling urinary catheters (77%), or mechanical ventilation (55%). Ninety-four percent of patients had wounds (i.e., surgical, pressure, or burn), and 61% experienced a recent or concurrent bloodstream infection. Seventy-four percent of patients were positive for *C. auris* clade I, and the other 26% were positive for clade III. Most (71%) patients were considered to have a clinical *C. auris* infection (vs. colonization) and received antifungal therapy.

**TABLE 1:** Characteristics of 31 patients with *C. auris* identified in the healthcare system (2021–2024)

Category	Measure
Demographics	
Male	65% (20)
Female	35% (11)
Age	59 years (range: 1–89 years)
Point of origin	
Outside hospital	18% (7)
Skilled nursing or long-term care facility	50% (19)
Other	32% (10)
Prior exposure to overseas healthcare facilities	6.5% (2)
Risk factors	
Stay in intensive care unit (ICU)	58% (18)
Wounds	94% (29)
Central venous catheter	74% (23)
Indwelling urinary catheter	77% (24)
Mechanical ventilation	55% (17)
Prior/concurrent bloodstream infection	61% (19)
Prior fluconazole use	32% (10)
Hospital days preceding first positive collection	10 days (range: 0–95 days)
Specimen source	
Multiple sources	35% (11)
Miscellaneous (wound, tissue, abscess, fluid)	55% (17)
Blood	42% (13)
Respiratory	29% (9)
Urine	29% (9)
*C. auris* clade	
Clade I (South Asia)	74% (23)
Clade III (South Africa)	26% (8)
Multiple clades	0% (0)
Treatment	
Yes	71% (22)
No	29% (9)
Antifungals used for treatment	
Micafungin only	45% (10)
Micafungin, amphotericin B	23% (5)
Micafungin, flucytosine	18% (4)
Micafungin, amphotericin B, and flucytosine	14% (3)

### Intra-patient and inter-patient *C. auris* diversity

**Fig 1:**
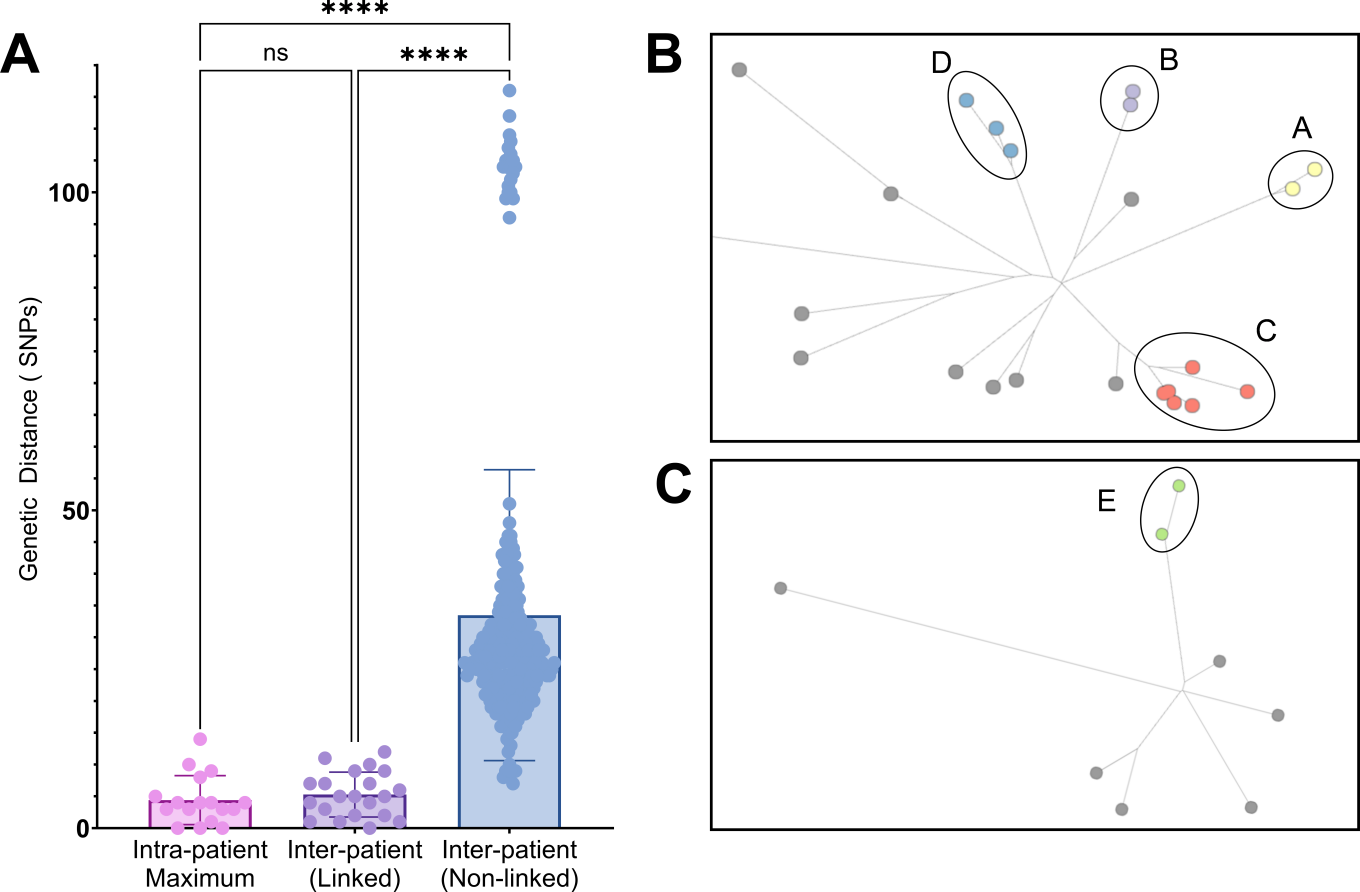
Comparison of *C. auris* genetic variation within and between patients in a healthcare system. (**A**) Intra-patient variation (pink) shows the maximum SNP distance observed between isolates from the same patient (*n* = 17). Using a single isolate from each patient, inter-patient variation within a clade was calculated. Data are categorized based on the presence (purple) or absence (blue) of epidemiologic links. Statistical significance is indicated (****, *P*-value < 0.0001; ns, not significant; Kruskal-Wallis, Dunn’s Multiple Comparisons test). (**B and C**) The Newick file output generated by the MycoSNP pipeline was used to construct a relatedness tree in MicroReact for Clade I (**B**) and Clade III (**C**). Enumerated circles (labeled A–E) and shades of the same color denote clusters of patients with epidemiologic links. One outlying Clade I isolate is not shown in the tree in panel B but is included in the SNP calculations in panel A.

Intra-patient *C. auris* diversity was analyzed in a subset of 17 patients for whom multiple *C. auris* isolates (range: 2–6) were available from unique sources and/or collection dates. The MycoSNP pipeline identified maximum intra-patient SNP distances ranging from 0 to 14, with a median of 4 SNPs (Fig. 1A; Table 2). Some patients had *C. auris* recovered from the same specimen type over extended periods, with SNP differences ranging from 4 to 9. For instance, CSF isolates from a patient (#9) varied by 4 SNPs over 73 days (Fig. S2). Blood isolates from a patient (#1) with persistent candidemia varied by 5 SNPs over 138 days, compared to 9 SNPs from a patient (#31) with recurrent candidemia 3 years after successful therapy. Additionally, a patient with cystic fibrosis (#15) and persistent respiratory colonization showed a 4 SNP difference between isolates collected over 309 days.

**TABLE 2:** Summary of allelic variation calculated by MycoSNP and refMLST^*a*^

	*n*	MycoSNP (SNPs)		refMLST (alleles)		Significance^*c*^
		Median	Range	Median	Range	
Intra-clade (Clade I)	23	27	0–48 (outlier: 116)	25	0–42 (outlier: 94)	*
Intra-clade (Clade III)	8	22.5	5–51	18.5	6–35	n.s.
Maximum intra-patient^*b*^	17	4	0–14	4	0–14	n.s.
Inter-patient (linked)	15	5	0–12	3	0–9	n.s.
Inter-patient (unlinked)	16	27	7–48 (outlier: 116)	25	6–42 (outlier: 94)	**

^*a*^ The MycoSNP pipeline calculates the number of SNPs, whereas refMLST additionally accounts for genomic insertions and deletions. One isolate per patient was used for calculations, unless otherwise indicated.

^*b*^ Based on multiple isolates from the same patient (ranging from 2 to 6).

^*c*^ Statistical significance was calculated via Kruskal-Wallis test (**, *P*-value < 0.01; *, *P*-value < 0.5; ns, not significant).

To assess inter-patient variation, one isolate from each of the 31 patients was included in a subset analysis (*n* = 31). Isolates were categorized as “linked” or “non-linked” based on the presence or absence of epidemiological connections, such as shared medical personnel/equipment (cluster C), referral from the same hospital (clusters B and D), or temporal overlap in hospital admission (clusters A and E) (Fig. 1B and C). In total, there were five clusters (comprising 15 patients) and 16 non-linked patients. Inter-patient SNP distances were compared within each clade. The median SNP distance among epidemiologically linked patients (median: 5 SNPs, range: 0–12) was significantly less than that between non-linked patients (median: 27 SNPs, range: 7–116) (Fig. 1A and *P*-value <0.0001; Table 2).

### Comparison of MycoSNP and refMLST results

Since the calculation of SNPs or alleles can vary based on the bioinformatic tool used, we analyzed the same data set using a turn-key commercial platform, refMLST (BugSeq, Vancouver, Canada). The maximum intra-patient distance calculated by refMLST was 14 alleles, consistent with MycoSNP (Table 2; Fig. S3A). For inter-patient variation, refMLST found a median distance of 3 alleles (range: 0–9) for linked patients and 25 alleles (range: 6–94) for non-linked patients. Significant differences between the two pipelines were observed only for non-linked patients (Table 2). When directly comparing distances generated by each pipeline, the linear regression model (y = 0.80X + 1.91) showed a statistically significant correlation (R^2^) of 0.81 (*P* < 0.0001) (Fig. S3B). Overall, clustering between the two outputs was similar (Fig. S3C and D).

### Length of stay prior to *C. auris* detection

**Fig 2:**
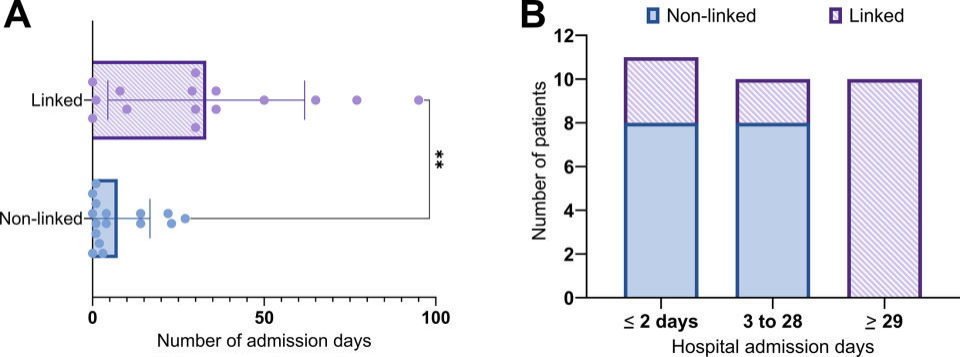
Number of hospital days preceding the collection of a specimen growing *C. auris*. (**A and B**) The number of hospital days preceding the collection of a specimen growing *C. auris* is shown, not accounting for prior admissions. Data are categorized based on the presence (purple, “Linked”) or absence (blue/solid, “Unlinked”) of epidemiologic links, and, in panel B, they are binned into positive collection occurring within 2 days, 3–28 days, or ≥29 days of admission. Statistical significance is shown (**, *P*-value < 0.01, Mann-Whitney test) in panel A.

To assess the likelihood that new *C. auris* cases were hospital-associated, we examined the number of inpatient days preceding *C. auris* detection, excluding prior encounters. The time to first detection was significantly greater for linked patients (median: 30 days, range: 0–95) compared to non-linked patients (median: 2.5 days, range: 0–27). (Fig. 2A and *P*-value <0.01; Fig. S2). Notably, all *C. auris* cases newly detected ≥29 days post-hospital admission were linked to a cluster (Fig. 2B; Fig. S2).

### Genotypic predictions of antifungal resistance

We analyzed 22 isolates with paired phenotypic susceptibility data to identify genotypic markers of resistance (Table S1). All isolates were fluconazole resistant (>256 µg/mL), with clade I isolates uniformly encoding a Y132F mutation in ERG11 but lacking the K143R mutation reported in other clade I isolates (20). All clade III isolates carried the ERG11 F126L and MRR1 N647T fluconazole resistance markers, with the latter conferring resistance via the upregulation of a drug transporter (21).

We observed phenotypic resistance to echinocandins, the preferred class of agents for treating *C. auris*, in an immunocompromised adult with chronic osteomyelitis (#7). The initial isolate from bone cultures had elevated MICs for fluconazole (≥256 µg/mL) and amphotericin B (2 µg/mL), but low MICs for the echinocandins (MIC = 0.5 µg/mL, micafungin and anidulafungin) (Table 3). After 2 months of micafungin therapy, the new isolate showed elevated echinocandin MICs (≥8 µg/mL). This phenotypic resistance coincided with the appearance of an F635C mutation in FKS1, the target for echinocandins (22). The resistant isolate was 9 SNPs apart from the initial, susceptible isolate.

**TABLE 3:** Phenotypic susceptibility testing and genotypic findings for a patient with *C. auris* osteomyelitis that failed micafungin therapy^*a*^

	Bone day 8	Bone day 74	Surgical wound day 74
Antifungal MIC (μg/mL)			
Fluconazole	**≥256^*b*^**	**≥256**	**≥256**
Amphotericin B	2	np^*c*^	2
Micafungin	0.5	1	**>8**
Anidulafungin	0.5	0.25	**>8**
Genotypic markers for echinocandin resistance	Absent	Absent	FKS1 F635C
SNP distance	np	0	9

^*a*^ Three *C. auris* isolates from the same patient were tested for antifungal susceptibility as part of clinical care. Minimum inhibitory concentrations (MIC) are shown for fluconazole, amphotericin B, and two echinocandins (micafungin and anidulafungin). SNP distances relative to the initial *C. auris* isolate are indicated.

^*b*^ Bold formatting is used to indicate drug resistance.

^*c*^ np, not performed.

**Fig 3:**
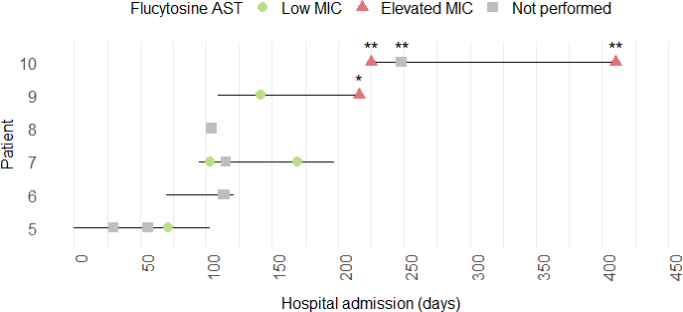
Flucytosine susceptibility within a *C. auris* transmission cluster. The hospital course for five patients (#5–10) linked to transmission cluster C is shown. The X-axis and solid horizontal lines denote hospital admission dates, and each node corresponds to a *C. auris* positive culture. Isolates are labeled according to the results of phenotypic testing for flucytosine susceptibility: low MIC (≤0.12 µg/mL, green circles), elevated MIC (≥32 µg/mL, red triangles), or susceptibility testing not performed (gray squares). Asterisks denote isolates where FUR1 mutations (*, G270R; **, Q16::STOP) were identified by WGS.

In transmission cluster C, we noted several isolates with elevated MICs (>32 µg/mL) to flucytosine (Fig. 3). Clonal isolates from serial CSF cultures in a patient with prolonged flucytosine exposure increased in MIC from 0.06 µg/mL to >32 µg/mL (#9, 4 SNPs; Fig. 3). Targeted examination of FUR1 and FCY1/2 loci, implicated in flucytosine resistance (23), identified a FUR1 G270R mutation. Another patient in this transmission cluster (#10), a neonate with prolonged hospitalization, showed consistently elevated MICs to flucytosine despite no recorded flucytosine exposure (Fig. 3). We hypothesized that the resistant isolates from patient #9 and #10 would carry the same mutations, given their close genetic relation (3–5 SNPs); however, WGS of an isolate from patient #10 instead revealed a FUR1 Q16::STOP mutation with no evidence of pre-existing G270R mutation.

## DISCUSSION

In response to national and regional increases in reported *C. auris* (24), we conducted a retrospective and prospective evaluation of cases within a healthcare system to assess patient risk factors, refine SNP or allelic thresholds for relatedness analyses, and identify genotypic markers of antifungal resistance. Most patients had recognized risk factors*,* such as ICU stays, use of invasive medical devices, and prior fluconazole therapy (25). International healthcare exposure was rare, suggesting that most cases in our health system were acquired in the United States. Previous studies in the Mid-Atlantic region identified similar risk factors and correlated outbreaks with socioeconomic vulnerability (26, 27).

Using epidemiologic and sequencing-based approaches, we identified five *C. auris* transmission clusters within the healthcare system. WGS is now a mainstay for assessing organism-relatedness during outbreak investigations (28). However, interpretation of SNP or allelic differences requires prior knowledge of inter- and intra-patient variation. In our study, we observed up to 14 SNP differences within a single patient, exceeding the 11 and 13 SNP differences reported by studies conducted in other geographic regions; however, those distances were calculated using different analysis pipelines or versions (29, 30). This possibly suggests a need for more relaxed thresholds for investigating healthcare-associated transmission. High intra-patient variability may result from prolonged *C. auris* colonization, allowing for genomic diversification over time, and our analysis of serial isolates provides some insight into expected genomic variation over time. The high variation may alternatively be due to independent acquisition events, but this scenario is less likely in regions with low *C. auris* prevalence. Our study found overlapping SNP ranges among transmission clusters (0–12 SNPs) and unlinked patients (7–116 SNPs), indicating potentially overlooked or indirect transmission events. Importantly, high genetic similarity does not confirm direct patient-to-patient transmission; patients within a cluster may be indirectly linked through multiple transmission events. It is therefore critical to interpret molecular findings within a broader epidemiologic context.

To identify healthcare-associated clusters of *C. auris* using the open-source MycoSNP pipeline, we propose stratifying relatedness into categories: very likely related (0–6 SNPs), possibly related (7–14 SNPs), and not closely related (≥15 SNPs). We propose similar ranges for the commercial BugSeq refMLST analysis: very likely related (0–5 alleles), possibly related (6–14 alleles), and not closely related (≥15 alleles). While the two pipelines revealed similar clustering patterns, the correlation between pairwise differences was not exact (R^2^ = 0.81). These findings exemplify the importance of validating interpretive thresholds for each bioinformatic tool, as previously noted (10), since SNP calculations can be impacted by factors such as the reference genome used and SNP filtering criteria.

The significant labor and reagent costs associated with sequencing may complicate an institution’s decision to perform WGS. In settings with low *C. auris* prevalence, sequencing of initial *C. auris* isolates from all new patients may be informative. Via this approach, we noted that all infections occurring more than 29 days post-admission were linked to a transmission cluster, representing a highly specific (100%) indication of healthcare-associated infection and a novel finding. However, most isolates included in this study were derived from clinical cultures with high suspicion for infection rather than colonization screens of asymptomatic patients. Given that *C. auris* colonization is expected to take place over a shorter timeframe (31) than progression to infection, the 1-month indicator may not be suitable for facilities that routinely screen for *C. auris*. A recent study estimated that 37% of U.S. facilities screen select patient populations for *C. auris,* either at admission or during point prevalence surveys following a positive case (32).

WGS also serves as a powerful tool for detecting and discovering genotypic markers of antifungal resistance. Our study documented the development of echinocandin and flucytosine resistance during therapy, a phenomenon previously reported for echinocandins that highlights *C. auris*’s adaptability and the growing threat of multidrug resistance (33, 34). In the presented case, WGS differentiated between two possible explanations for echinocandin treatment failure—the *de novo* emergence of resistance versus the acquisition of a new *C. auris* strain—a distinction critical for directing outbreak investigation resources efficiently. Additionally, our study identified two putative FUR1 mutations (G270R and G16::STOP) that may confer flucytosine resistance and warrant further characterization. The mechanisms conferring resistance to other antifungals (e.g., amphotericin B, flucytosine, and new agents like Ibrexafungerp) remain incompletely understood; however, paired WGS and phenotypic susceptibility data can aid the discovery of novel resistance markers.

In summary, our findings provide interpretive thresholds for *C. auris* relatedness across two bioinformatic tools and illustrate the utility of WGS in identifying transmission clusters and genotypic markers of resistance. We recommend that institutions with low-to-moderate *C. auris* prevalence consider implementing WGS to support outbreak investigations and inform infection control strategies.

## Data Availability

The sequence data generated in this study (accessions SRX32028011–SRX32028078) have been deposited in the NCBI Sequence Read Archive (SRA) under BioProject ID PRJNA1418105 and are publicly available at http://www.ncbi.nlm.nih.gov/bioproject/1418105.

## SUPPLEMENTAL MATERIAL

Supplemental figures and table jcm.01348-25-s0001.pdf Figures S1 to S3; Table S1.

ASM does not own the copyrights to Supplemental Material that may be linked to, or accessed through, an article. The authors have granted ASM a non-exclusive, world-wide license to publish the Supplemental Material files. Please contact the corresponding author directly for reuse.
