# Supplementary material for: Detecting healthcare-associated transmission and antifungal resistance in Candida auris via whole genome sequencing
Source: J Clin Microbiol. 2026 Mar 25;64(5):e01348-25. doi: 10.1128/jcm.01348-25 (PMC13170312; doi:10.1128/jcm.01348-25)
Supplement: Supplemental figures and table — Figures S1 to S3; Table S1. [file jcm.01348-25-s0001.pdf]

Supplementary Figure S1. Summary of *Candida auris* isolates analyzed by Whole Genome Sequencing. Sixty-eight *C. auris* isolates from 31 patients were included in the analysis. (**A**) Isolates were derived from clinical specimens collected between 2021 and 2024. (**B**) Specimen source/types included blood, respiratory, wound, urine, tissue, fluid, CSF, abscesses, and surveillance swabs of the axilla/groin. (**C**) Whole genome sequencing was performed on Illumina platforms (see Methods section) and the median sequencing depth for isolates is shown. **(D)** The Newick file output generated by the MycoSNP pipeline was used to construct relatedness tree in MicroReact. Nodes correspond to isolates from Clade I (green), Clade III (orange), or the reference (gray).

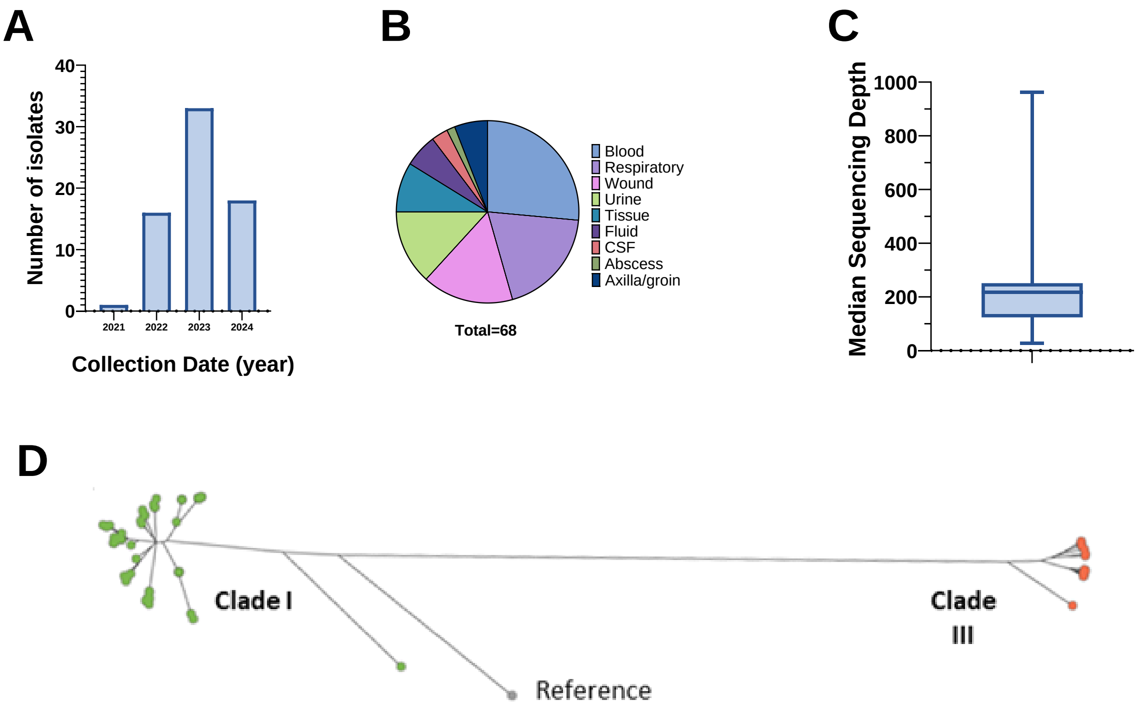

Supplemental Figure S2. Timeline for *C. auris*-positive cultures relative to hospital admission date. Patients and assigned transmission clusters are denoted with numbers and letters, respectively. Each node indicates collection of a *C. auris*-positive specimen and node color indicates source (blood, red; CSF, purple; tissue/fluid/abscess, pink; wound, orange; respiratory, blue; urine, yellow; axilla/groin, gray. Vertical dashed line at day 29 represents the threshold after which new *C. auris* detections were uniformly tied to a transmission event. Asterisks indicate isolates where SNP distances are noted in the text: Patient #1, 5 SNPs; Patient #9, 4 SNPs; #15, 4 SNPs; #31, 9 SNPs.

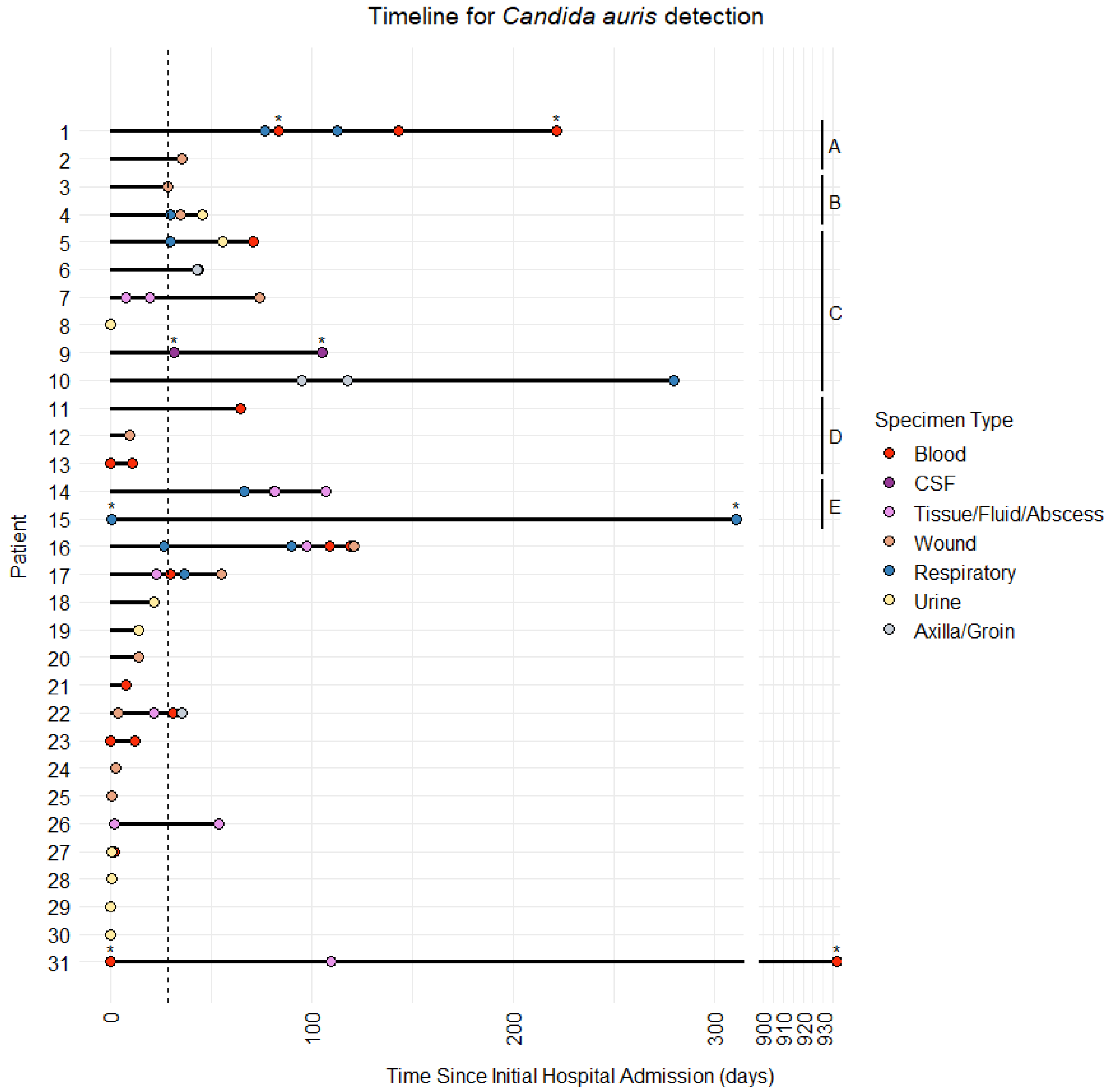

Supplementary Figure S3. Comparison of *C. auris* genetic variation using refMLST. (A) Intra-patient variation (pink) is showing the maximum allelic distance observed between isolates from the same patient (n=17). Inter-patient variation was a calculated using a single isolate from each patient and is categorized based on the presence (purple) or absence (blue) of epidemiologic links. Statistical significance is indicated (****, P-value < 0.0001; ns, not significant; Kruskal-Wallis, Dunn's Multiple Comparisons test). **(B)** Unique SNP distances calculated with MycoSNP (x-axis) are plotted against allelic distances calculated by refMLST (y-axis). A linear regression model was fit to the data (y=0.80X + 1.91) with a significant coefficient of correlation (R^2) of 0.81 (p < 0.0001). **(C-D)** Distance matrices were used to construct relatedness trees for Clade I **(C)** and Clade III **(D)** in MicroReact. Circled nodes represent epidemiologically defined clusters of patients. One outlying Clade I isolate is not shown in the tree in **(C)** or included in **(B),** but is included in (**A**).

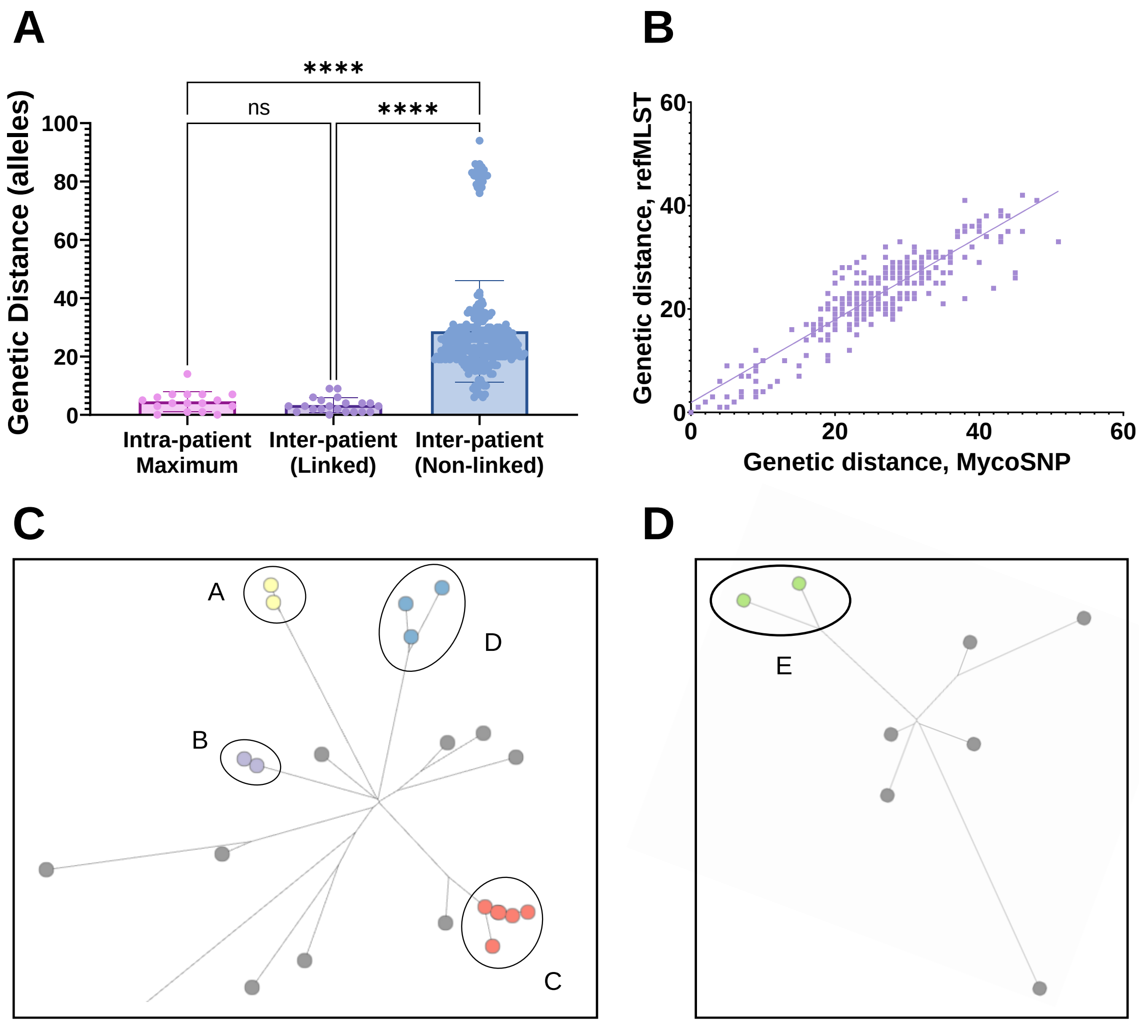

Supplementary Table S1. Antifungal resistance of *C. auris* isolates across the hospital system. Twenty-two isolates with paired phenotypic susceptibility testing results were categorized as susceptible or resistant using tentative minimum inhibitory concentration (MIC) cut-off values recommended by CDC. Detection of known genotypic markers of antifungal resistance is noted for each clade.

**Antifungal**	**MIC cut-off values**	**Resistance (n)**	**Genotypic Markers**	
			Clade I	Clade III
Fluconazole	≥32 µg/mL	100% (22)	ERG11 Y132F	ERG11 F126L, MRR1 N647R
Micafungin	≥4 µg/mL	4% (1)	FSK1 F365C	N/A
Flucytosine	N/A (≥32 used)	9% (2)	See Fig 3.	N/A
